# Achieving long-term success in irrigation commons

**DOI:** 10.1371/journal.pone.0353875

**Published:** 2026-07-21

**Authors:** Ulrich J. Frey, Atul Pokharel

**Affiliations:** 1 Department of Environmental Systems Sciences, University of Graz, Graz, Austria; 2 Department of Computer Science, Yale University, New Haven, Connecticut, United States of America; Universidad de Murcia, SPAIN

## Abstract

Which characteristics predict the long-term performance and persistence of irrigation systems? This question has been challenging to answer due to the lack of longitudinal data. Numerous cross-sectional studies have identified a wide range of relevant factors. However, it is unclear which of these factors remain associated with long-term persistence, or what their relative predictive power is. To examine performance and survival after a period of 16–37 years with respect to concerning initial system attributes, we have created a unique longitudinal dataset of 218 community-based irrigation systems in Nepal, separated by three decades. We applied the best available models using several state-of-the-art machine learning algorithms to ensure method independence. Our findings show that farmer-managed systems that receive external support and whose leaders are users perform best. Long-term survival is associated with fair rules, regular member meetings, and occasional external assistance. Good institutional design is consistently among the strongest predictors of long-term performance and persistence in irrigation commons.

## Introduction

Commons resources are central to addressing global hunger and the needs of a growing human population. Irrigation systems lie at the core of food production and increasing demand will put further strain on Earth’s limited water resources. Irrigated agricultural systems account for almost 90% of global water consumption and contribute to about 40% of all crops [1]. Furthermore, a large proportion (90%) of agricultural production takes place in small-scale, local systems [2]. Many of these community-managed systems are common property regimes [3], accounting for an estimated 65% of Earth’s surface [4]. Thus, commons-based irrigation management is essential to achieving sustainable development goal number two, zero hunger.

Successful governance of common pool resources, including forests, fisheries, and irrigation systems, is crucial for the sustainable use of the Earth’s resources [5]. Starting with the *design principles* of Elinor Ostrom [6,7], various approaches tried to identify key success factors [8,9]. These approaches have identified various institutional factors as important [10–12] and a smaller set of factors have been commonly emphasized across studies. These include participation of users, monitoring, clear property rights, and dependency on the resource [8,12,13].

However, a causal theory linking these factors is lacking, and potential factors have not yet been organized into a hierarchy based on relative significance. Moreover, many institutional characteristics are already endogenous at the time they are first observed. Consequently, even when early measurements precede later outcomes, observational data generally do not support causal interpretation. This paper therefore does not attempt to identify causal effects. Instead, we adopt a predictive perspective, asking the question: How can we predict long-run outcomes?

This means that, while any given aspect of institutional design may be significant, sometimes it may have opposite effects in different instances [12,13]. For example, it may be positive if the leader of the commons user group comes from the group itself because of their knowledge and commitment [14]. However, they may also become corrupt and exploit the system for personal gain, which is clearly negative. The interrelationships of institutional factors are recognized to be complex, and there is little agreement in the literature on which characteristics are associated with the performance of irrigation systems. It is clear, however, people prefer institutional designs to non-institutional settings, behaving more cooperatively there [15]. People are also highly context-sensitive [16–18], exhibiting varying cooperation rates across cultures and subject pools [19–21].

Despite much research having been conducted into public goods [22,23], field experiments [24–26], numerous case studies [11] and a few larger databases for forestry, fisheries, and irrigation systems [6], the fundamental question of which factors are associated with the long-term success of community-managed irrigation systems remains largely unanswered [27,28].

This gap persists due to three main problems: first, the difficulty of generalizing findings from individual case studies, second, the complete lack of longitudinal data for irrigation systems, and third, the lack of comprehensive datasets with a common methodology [8,29]. As a result, previous research has not reached robust conclusions [8]. The same is true of meta-analyses of community-managed fisheries [9,30] and forestry [12,31].

This paper addresses the first (single case studies) and second problem (lack of longitudinal data) by creating a large (n = 232) unique longitudinal dataset of irrigation systems in Nepal, using an existing data set from Elinor Ostrom as round 1 and collecting a new data set as round 2. Since variables are collected consistently and follow the same methodology, the SES (Social-ecological systems) framework [32], this approach addresses the third problem (lack of standardization), where each case study conceptualizes success factors differently and expresses them through different variables [33].

Our approach allows, for the first time, to quantitatively characterize the long-term performance and persistence of irrigation systems. For example, it is now possible to examine how past characteristics relate to current performance, and to compare the relative predictive power of factors over time, including whether institutional design is associated with survival. Given the aforementioned critical role of irrigation systems in global nutrition, with 70% and 50% of China’s and India’s food production, respectively, coming from irrigated areas [34], considerable effort has been invested in improving their performance [35,36]. Existing research suggests that the participation of users and working institutions are particularly relevant [8,37,38].

However, these studies provide only snapshots and give limited insight into long-term dynamics [39]. With only a few large, longitudinal datasets available in commons research at all [40], revisiting the Nepal Irrigation Institutions and Systems (NIIS) dataset, created by Elinor Ostrom, has allowed us to create a unique longitudinal dataset for irrigation systems. This dataset stands out in its size and internal validity [29].

In addition to using a large N longitudinal dataset, this study also addresses methodological problems. Two key methodological issues in the literature are the persistence of regression analysis, despite evidence that machine learning could considerably improve the model performance for irrigation data [8], and the wide variation in estimates of variable importance between methods [41]. To overcome these challenges, this study uses multiple models and compares their variable importance to achieve more robust results to answer the following research questions:

1. Why do some irrigation systems survive and others do not?
2. What makes some systems successful and why do others fail?

Despite decades of sustained effort and broad recognition of their significance, these fundamental questions remain largely unanswered. We help answer these questions by addressing two major challenges. First, by creating a unique longitudinal dataset for irrigation systems with strong internal validity, we enable long-term prediction of success. Second, we devise a rigorous machine learning pipeline using ten algorithm variants that represents a significant improvement both in prediction precision and robustness compared to the widely used regression analyses.

## Data

The original dataset consists of 263 irrigation systems in Nepal collected in the late 1970s into the 1980s by Elinor Ostrom and her team. The irrigation systems in this sample are geographically diverse, covering all three major geographic regions (plains, mid-hills, high mountains). The systems vary in size from 10 to 6000 users and 2–8700 hectares as well as management regime, but the original site selection in the 1980s did not follow strict criteria, hence may not be representative. However, 30 out of 75 administrative districts in Nepal are covered (S1 Fig. S2 in S1 File). The NIIS dataset is very well-known and widely studied in the literature [8,27,42].

The second author completed a second round of data collection (R2) on these systems in 2013 using the same set of survey instruments. Therefore, the dataset includes two rounds of data, with a gap of 16–37 years between observations, because the first dataset was encoded from case studies that were completed between 1976 and 1998.

Round 2 followed the methodology of the first round (R1) for methodological consistency. Both datasets have been made openly available by the authors (https://osf.io/4yvj7/overview). For much more information on the systems, survey methodology, sampling, coding of variables, and protocols, see Appendices 1 and 2 of [43]. Each system contains information on about 500 variables, making it one of the most complete datasets existing in SES research on the case study level with a high internal validity [29].

This study focuses on elucidating the performance of these systems in 2013 by examining their state 30 years earlier (R1 explaining R2). Additionally, the study includes analyses of internal consistency, with cross-sectional analysis (the first round of data collection explaining the success of R1 and the R2 variables explaining R2).

The original data (R1) focuses primarily on institutional design and contains limited ecological information. Therefore, we supplemented it with rainfall data from ERA5, as detailed in the supplementary material (S1 Section 1). The independent variables are binary, and correlations are only calculated for the numerical variables (i.e., various size measures and rainfall data). Each of these shows high correlations (see S1 Section 5 and S1 Fig. S1 in S1 File).

Since machine learning algorithms require complete data for each case study, some of the variables associated with success required imputation (see S1 Section 2). These data points were missing completely at random (MCAR), and correlational analyses did not reveal any patterns in the missing data. The sources of missing data varied across items in both R1 and R2. These included instances where respondents lacked sufficient knowledge to provide an answer, cases involving inherent ambiguity, and situations where subjective disagreements among team members could not be reconciled through our established protocol (described in S1 Section 12).

Before imputation, *variables* with more than 60% of missing data were excluded. This threshold was chosen since imputation quality of variables dropped significantly if more than 60% were missing. After that, *cases* with too much information missing (> 40% of variables) were also excluded prior to imputation. Imputation was performed using the widely used R package *Mice* [44] for handling missing data. It creates multiple plausible datasets by imputing missing values through iterative regression models. After analysis, results from these datasets are combined to produce unbiased estimates. This multiple imputation employed additive regression, bootstrapping, and predictive mean matching. These results were cross-validated, using different imputation methods by the *Hmisc* package [45]. Pearson correlations of imputed values between methods range between 0.97 and 0.99, indicating a near perfect agreement. The final imputed dataset consists of 218 out of 263 systems with 28 R1 and 28 R2 variables. All data preparation, recoding and analyses were performed using *R Statistical Software 4.3.1* [46].

Success is measured by seven performance variables. Three variables describe the situation at the tail end, namely adequacy (water supplied is sufficient to meet crop water needs), predictability (timing and amount of water supplied are reliable and expected), and equity of water distribution (no/yes) [27]. Two describe the maintenance of headworks and canals (poor, fair, good) and two capture whether there is deterioration of canals or the headworks (yes/no). Finally, we record R2 survival (yes/no). Table 1 presents the summary statistics for these performance indicators.

**Table 1:** Summary statistics of performance variables (no = 0, yes = 1; poor = 1; fair = 2; good = 3).

Variable name	n	mean	sd	min	max
**Round 1 (R1)**					
headworks: how well maintained (poor, fair, good)	218	2.05	0.67	1	3
canal: how well maintained (poor, fair, good)	218	2.13	0.69	1	3
deterioration in canal (yes/no)	218	0.32	0.47	0	1
deterioration in headworks (yes/no)	218	0.28	0.45	0	1
predictability of water at tail (no/yes)	218	0.80	0.40	0	1
adequacy of water at tail (no/yes)	218	0.40	0.49	0	1
equity of water at tail (no/yes)	218	0.72	0.45	0	1
**Round 2 (R2)**					
headworks: how well maintained (poor, fair, good)	218	1.89	0.84	1	3
canal: how well maintained (poor, fair, good)	218	1.85	0.90	1	3
deterioration in canal (yes/no)	218	0.71	0.46	0	1
deterioration in headworks (yes/no)	218	0.56	0.50	0	1
predictability of water at tail (no/yes)	218	0.81	0.39	0	1
adequacy of water at tail (no/yes)	218	0.69	0.46	0	1
equity of water at tail (no/yes)	218	0.68	0.47	0	1
system survived (no/yes)	218	0.88	0.32	0	1

## Methods

### Survey methodology

Multiple trained coders with many years of experience implemented the methodology for the first-round survey. The second round (R2) follows this method for consistency reasons. All persons administering the survey were trained for 2–3 weeks and had a college education. Each team consisted of two members, at least one of which was a civil engineer.

At least three users answered the questionnaire. The first person was a user who was willing to answer questions. The second person is someone who was pointed out by others as being very knowledgeable about the irrigation system. The third informant was someone who was economically underprivileged on average, as determined by plot size and the condition of their assets. If one of the respondents answered “poor” to a question about a physical condition, the system was coded as poor in that dimension. For all other answers, the most frequent response was entered into the database and group interviews were also conducted. The performance variables of water supply adequacy, predictability and reliability at the end of the process are based on the respondents’ answers. The survey teams were trained to assess these metrics consistently.

The sampling method, distribution and characteristics of irrigation systems, and survey methodology are described in length in [43]. Further information about the data collection, summary statistics about all variables, e.g., size, who built these systems, etc. can be found in the supplementary materials (S1 Table S13 in S1 File).

### Machine learning

Machine learning algorithms typically outperform typical statistical approaches like multivariate linear regressions on predictive performance. This is especially true for this particular data set, since one study has shown through a direct method comparison [41] that predicting out-of-sample success of for these irrigation systems works much better with machine learning approaches. Therefore, we use these state-of-the-art algorithms for the best predictive model performance.

However, even very similar algorithms such as random forests and gradient boosting sometimes yield conflicting results about the significance of variables [47]. Therefore, for robust conclusions, we compare 5 different algorithms – decision trees, random forests, gradient boosting, general linear models and support vector machines, both tuned and “untuned”, i.e., with the default parameters, serving as benchmark (S1 Section 3 in S1 File), resulting in 10 variants. All algorithms were implemented using the R package MLR3 [48]. These algorithms were chosen for their superior performance and we provide the machine learning code in the supporting material. The models were tested for accurate classification of the seven performance variables listed in Table 1. The list of independent variables can be found in the supplementary materials (S1 Table S1 in S1 File). In addition, analyses were performed using the H2O package [49] to rule out any possible model artifacts resulting from the package implementation. No such artifacts were detected.

Training was performed on 80% of the data sets, with 10% held back for three-fold cross-validation and the remaining 10% used for testing. Due to the small data set size, we opted for 80% instead of 70% [41]. The test set, which the algorithm does not see, was then used to evaluate prediction accuracy, expressed as the percentage of correct classifications (i.e., deterioration or no deterioration). A different metric (log loss) was chosen for the survived variable, since accuracy is not suitable for highly skewed variables (88% of systems survived). Log loss is a measure of how well predicted probabilities match the true outcomes, penalizing confident wrong predictions more strongly.

Both a random grid search method and Bayesian optimization were used for hyperparameter tuning, as these have been shown to be superior to other tuning methods across various domains. Each algorithm has parameters that require tuning, and the ranges for these are provided in the supplementary materials (S1 Section 3 in S1 File). However, the default parameters often proved to be on par with their tuned counterparts. To ensure comparability between the machine learning algorithms, the duration of each search was set to the same value for each run. Search times varied between 10 and 150 minutes. For decision trees, gradient boosting and random forests, the relative importance of each variable was extracted.

With a multitude of variables and models, spurious correlations become more likely. It is therefore important to note that the variables included in our analysis were strictly those identified by prior studies and theory as potentially significant for survival and performance over time. Due to the number and complexity of these variables, we refer to [33] where all these factors are defined, their significance and pathways for success, and their relationships are described in detail. Thus, our study can be understood as measuring the relative significance of factors hypothesized to be important for long-term survival. One limitation of this approach is that we miss the opportunity to identify novel factors, despite having constructed a thorough longitudinal dataset. However, we also reduce the possibility of identifying spurious factors. We were also able to identify the machine learning models and algorithms that performed best for this particular application.

## Results

### Predicting performance from data 30 years ago

In line with previous research [27,50], we consider survival and performance. When analyzing performance, we distinguish between the physical condition of the headworks and the canals, and between tail equity as well as tail adequacy (Table 2). However, unlike existing studies, the following results predict performance in 2013 using just the data from 30 years ago. Hence, long-term results (LT) are not comparable to snapshot studies.

**Table 2:** Best models for each of the six performance variables.

Performance variable	Model	Train time (minutes)	Model type	Accuracy
Maintenance of headworks(poor, fair, good) DP2	SVM tuned	60	institutional factors only	LT = 0.59 R1 = 0.55 R2 = 0.57
Maintenance of canals(poor, fair, good) DD2	SVM tuned	60	institutional factors only	LT = 0.67 R1 = 0.59 R2 = 0.73
Deterioration of headworks(poor, fair, good) P1	DT not tuned	40	all	LT = 0.77 R1 = 0.70 R2 = 0.80
Deterioration of canals(poor, fair, good) D1	DT not tuned	15	all	LT = 0.89 R1 = 0.86 R2 = 0.89
Predictability of water at tail end(no / yes)	many	20	all	LT = 0.92 R1 = 0.91 R2 = 1.00
Adequacy of water at tail end(no / yes)	GBM tuned	50	institutional factors only	LT = 0.84 R1 = 0.82 R2 = 0.89
Equity of water at tail end(no / yes)	SVM not tuned	15	all	LT = 0.86 R1 = 0.82 R2 = 0.93

Note: LT = Predicting R2 performance (2013) from R1 data 30 years ago; R1 = Predicting R1 performance from R1 variables; R2 = Predicting R2 performance from R2 variables; Model type “all” also contains number of users and main canal length.

Across the board, both untuned Decision Trees and tuned Gradient Boosting perform best, but there is no clear overall winner. The sweet spot for training time seems to be around 40–60 minutes – models trained for three hours do not perform better, possibly due to overfitting.

None of the models for a specific period, whether R1, R2 or LT, perform consistently better across all variables. Thus, models for 30 years reach about the same accuracy as models for either round 1 or 2.

The most difficult performance variables to predict are *maintenance of headworks* and *maintenance of canals*. This is plausible, since these depend on the time the systems have been surveyed – repairs might have been scheduled, but not yet realized. Here, the best models correctly predict 67% (SVM) and 59% (SVM) of all cases, respectively. It is comparatively easier to predict the *deterioration of canals* (89%, DT) and *headworks* (77%, DT).

In contrast, *tail adequacy* (84%, GBM), *equity* (86%, SVM) and *predictability* (92%, many models) can be predicted rather accurately, even with data from 30 years ago. Given that the majority of schemes have indeed survived, and that institutional design changes slowly in successful schemes because there is little need, this seems plausible.

The causal pathways between influencing factors and outcomes are complex [33]. For example, a high participation rate can lead to quick and targeted adaptation of rules. In turn, a high dependency on the resource system increases the likelihood of participation, e.g., in user associations. Tail equity relies on fair and open processes which benefit from high participation levels.

### Comparing model performance across algorithms

**Fig 1:**
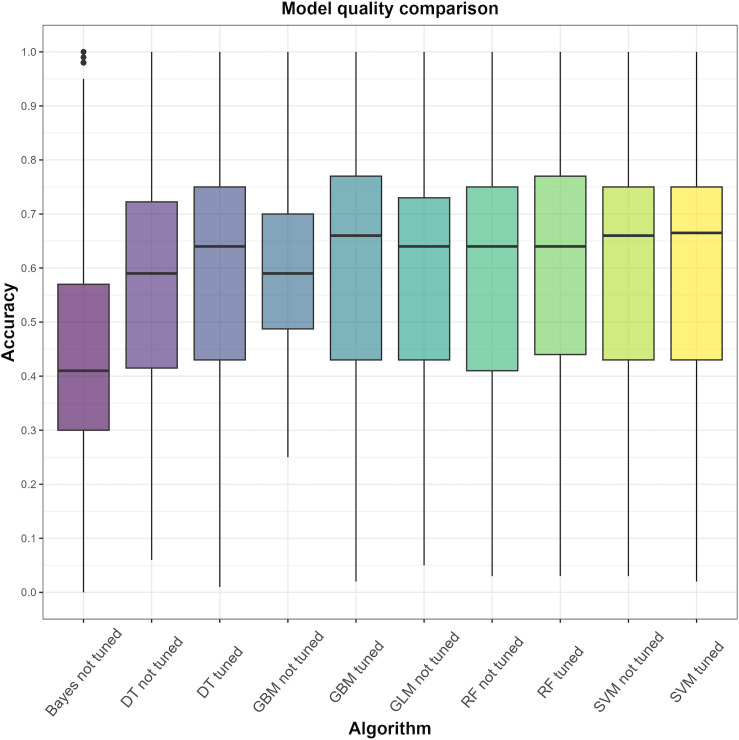
Accuracies across machine learning algorithms and all performance variables in both R1 and R2 (higher = better predictive accuracy).

Fig 1 shows an overview of the accuracy of all model runs for the test sets (= out-of-sample) across all seven performance variables (for details, see S1 section 4 in S1 File), without delving into the importance of contributing factors (see section 1.4 and Table 1.3 for that).

Median accuracy across all seven performance variables is relatively stable at about 0.65, except for the Bayesian models. The two tuned gradient boosted models (XGBoost) perform slightly better than the others. In each case, the tuned models outperform their untuned counterparts, albeit sometimes only slightly. This consistent performance indicates that a large number of models accurately estimate the importance of contributing factors.

The mean accuracy is highest (0.75) for *predictability at the tail*, and lowest for *maintenance of the headworks* (0.39). The best models (see Table 2 below) are fairly accurate, often classifying systems with 80–90% accuracy. ML-results always have to be compared to a benchmark and in this case, benchmark models are featureless, i.e., they always predict the majority of the cases. In the case of skewed outcomes, e.g., survival, where 88% of systems survive, this makes it especially hard to outperform them, since they predict survival correctly 88% of the time. In general, benchmark models are often only a few percentage points below the best ML-models.

### Survival

Can we predict 30 years in advance which systems will survive? This would be of immense value for the design of irrigation systems. It would also come as no surprise if this were not possible, given the long time and – in this case – the many major external changes in Nepal like war or revolutions. Yet, some models predict survival rather precisely. Here, survival refers to whether a canal was in use at the time of the second survey. A system that falls into disuse but is then revived and becomes functional once again is still considered a surviving system if the users are largely the same and the system was used at the time of the second survey.

As the sample is highly skewed, with 88% of all systems surviving, accuracy is not an appropriate measure of error. Instead, log loss is used. The best models for predicting system survival are those that include only institutional design variables. All models that include system size (area and number of users) are worse by ~ 6% in accuracy and 0.05 in log loss. Correlations between the number of users and the length of main channel are only moderately strong (see S1 Section 5). This is likely because of variations in geography – long canals in hilly areas typically serve fewer users than in the plains.

The best algorithm (tuned decision trees) predicts survival with a log loss (error of prediction probability) of 0.17 and is thus better than the baseline featureless benchmark model with a log loss of 0.23.

### Which factors are most predictive of performance and persistence?

There is little agreement in the literature on which characteristics are associated with the performance of irrigation systems [39]. There are several reasons for this. On the one hand, differences may be due to factors being conceptualized, measured and/or modeled differently [33]. In addition, the choice of method itself plays a role, as machine learning algorithms assign quite different importance to variables, even when using the same dataset [47].

A major contribution of this paper is its ability to address these issues. First, only results that are robust across ten different machine learning algorithms are reported. Second, seven performance variables, drawn from existing studies and theory, are used to capture nuances in performance. Third, three explanatory models (R1, R2 and LT) complement each other (see S1 Tables S3 and S4 in S1 File). Fourth, we distinguish between three sets of variables: one set considers only institutional variables as independent variables; one set considers only physical variables (e.g., number of users, system area or rainfall); and one set considers all variables (see S1 Tables S3 and S4 in S1 File). This separation allows the stability of the influence to be estimated more accurately across different sets of variables. Finally, the second-round data (R2) were collected using the same protocols as the first (R1) round to maintain the internal validity of the concepts and their measurement.

We calculated the importance of each performance variable (see Table 3) and survival across all models (n = 218). The three factors with the highest mean per performance variable across machine learning models are reported. Therefore, variables that are consistently associated with success in the majority of these variations should be highly relevant to a large number of irrigation systems in general.

**Table 3:** The three most important variables for LT (relative factor importance) with directions in parentheses.

Performance variable	Importance	Mean %
*Maintenance of headworks*	1. Are penalties enforced? (enforcement = better) 2. Who enforces the rules? (community involved = better) 3. Who constructed the system? (community involved = better)	**1.** 5.72 **2.** 5.50 **3.** 3.00
*Maintenance of canals*	1. Is the leader position filled by takers? (community involved = better) 2. How is the leader selected? (community involved = better) 3. Have the takers ever tried to change their rules? (change = better)	**1.** 10.26 **2.** 7.30 **3.** 4.94
*Deterioration of headworks*	1. Has any agency or government given assistance? (assistance = better) 2. How big is the difference between the wealthiest and poorest members? (equality = better) 3. Is there considerable variation in the flow of units from year to year? (less variation = better)	**1.** 16.12 **2.** 10.85 **3.** 8.47
*Deterioration of canals*	1. Has any agency or government given assistance? (assistance = better) 2. How long have the rules-in-use been in place? (longer = better) 3. Does that group operate the headworks alone? (community involved = better)	**1.** 23.57 **2.** 11.79 **3.** 9.72
*Predictability of water at tail*	1. How frequently do most takers use these arenas to discuss mutual problems of the resource? (more frequent = better) 2. What percentage of members spends a lot of time in non-agricultural activities? (less = better) 3. Who enforces the rules? (community involved = better)	**1.** 14.97 **2.** 8.39 **3.** 7.17
*Adequacy of water at tail*	1. Has any agency or government given assistance? (assistance = better) 2. Does the leader report to any external or higher-level authority? (yes = better) 3. In the past few years, have takers been taking more or less from the canal than before? (less = better)	**1.** 12.54 **2.** 10.50 **3.** 7.22
*Equity of water at tail*	1. Are there arenas being used for the exchange of information about conditions of the resource? (yes = better) 2. How long have the rules-in-use been in place? (longer = better) 3. Is there a penalty (for rule-breaking)? (yes = better)	**1.** 13.07 **2.** 10.91 **3.** 7.47
*Survival*	1. Are the rules-in-use perceived by takers as fair? (fair = better) 2. Has any agency or government given assistance? (assistance = better) 3. Are there arenas being used for the exchange of information about conditions of the resource? (yes = better)	**1.** 10.61 **2.** 8.45 **3.** 8.16

The following section discusses the main influences for each performance variable.

The *condition of the headworks* of irrigation systems is related to outside assistance with penalties that are enforced, and only a small perceived economic gap between rich and poor members. Participation of users in the decision-making process also shows a strong predictive association with success, likely because it is a critical factor for rule enforcement [12,51]. However, the many possible causal interrelationships between factors are not the topic of this analysis (but see [33,52]) and have not been modelled quantitively yet.

For the *canals*, outside assistance by the government or NGOs and stable rules are equally predictive for success. Systems in which users themselves are in charge — including construction, operation of canals, and management — tend to show higher persistence.

*The predictability of water* at the tail end is associated with good communication in arenas where problems can be discussed. Frequent user meetings and fair, user-adjustable, user-enforced rules are likewise associated with survival. It is also relevant that not too many members spend time in non-agricultural activities.

For *water adequacy* at the tail end, the relevant factors are outside support, but also that users can manage their system themselves. It is also relevant that users do not take more than their share water from the canals than before.

Stable and fair rules are a precondition for *equity of water* at the tail. Again, this is a good validation of our models, as these variables are expected to be closely related. The presence of frequently used dispute-resolution arenas and of penalties is also associated with better outcomes. Perceived rule fairness is among the characteristics most strongly associated with persistence. Outside assistance in critical periods is also important. In addition, it is critical that the users can manage the system themselves and address the rules according to changing needs by using appropriate arenas.

Overall, interventions from non-governmental organizations or government agencies show among the strongest associations with performance and persistence in our data. Having a leader from within the group, regular meetings, and stable rules are also among the most predictive characteristics overall [53,54]. A wide gap between poor and rich group members is detrimental to success, and enforcement is positively associated possibly because good institutional design only matters if rules are enforced [6,7].

Surprisingly, enhancing the system or avoiding harm to it seems not to be particularly relevant for success. Physical variables like variation of water over time, for example due to rainfall, have some short-term relevance, but do not play a major role long-term. Penalties and their enforcement are important in some models, but not in others.

A comparison with the design principles of Elinor Ostrom [6] finds that three design principles – graduated sanctions, collective-choice arrangements, and minimal recognition of rights to organize – play the most important role if matched to the performance variables. A detailed table can be found in the supplementary materials (S1 Table S14 in S1 File). This ties in with the work of Cox et al. [7] who also find support for these design principles while pointing out that other important factors are missing, like fairness in our case.

Finally, we remain unsure about the role of system size measured by the number of users and the length of canals (see S1 Section 5 in S1 File). On the one hand, when included in the models, size dominates the importance ranking, like in community forestry [55]. On the other hand, if left out, models are about the same quality. This is in line with previous results with this dataset, which also found no clear effect of size [50]. A dedicated study is needed to tease these complex relationships apart.

### Successful versus unsuccessful systems

In the dataset, there is a wealth of information on what is characteristic of stable, well performing versus unstable or failing systems. Since five of the seven performance variables are binary, they naturally fall into two groups that can be tested for differences with t-tests. The two variables for the maintenance status of the headworks and the canals are transformed into binary ones by lumping “good” and “fair” together as opposed to “poor”. All reported tests in the text are significant at the 0.05 level with Bonferroni correction. The exact means per group, t-values and degrees of freedom can be found in the supplementary materials (S1 Section 7 in S1 File).

*Good maintenance of headworks and canals* is related to the availability of arenas for users to meet and discuss, equity and adequacy of water distribution at the tail end, a decreasing water use rate, and interventions by the government or NGOs.

*Deterioration of headworks and canals* is associated with less maintenance, and no penalties. Deterioration is also less severe if there is no other canal available as an alternative and if users can participate in system governance.

*Predictability* of the waterflow at the tail is associated with less variability of water over time, is better if the system is built by the government or NGOs (instead of the users themselves), and, surprisingly, surviving systems have less predictable flow at the tail end. It is independent of other performance variables.

More *equity* at the tail end is coupled with much higher maintenance of headworks and canals, a decreasing use rate, no alternatives, and better enforcement of the rules.

An *adequate* water supply is also tied to decreasing use, equity, good communication between government and water user associations, and good maintenance of the physical hardware.

Finally, *survival* is related to having established penalties, predictability of the water supply, and only small differences between the rich and the poor families of the group.

## Discussion and conclusion

By analyzing a longitudinal dataset with several ML algorithms, classifying systems into successful and unsuccessful systems can be done robustly for most performance indicators with a rather high accuracy. Long-term persistence is strongly associated with indicators of good institutional design observed 30 years earlier, consistent with conclusions from the literature that early warning signs are apparent before a collapse [56]. Both autonomy in day-to-day decision-making and external assistance support success and survival. This is backed up by previous studies [33,43,50]. Some patterns may be obscured by the time between rounds, which included a wide range of shocks, e.g., civil war with two new constitutions, a change from monarchy to a federal republic, mass labor migration, and urbanization.

There are some differences between algorithms, most notably the well-known fact that the time requirements differ substantially across algorithms. However, for our field of study this is deemed as relatively unimportant, since all calculations can be performed on a standard desktop PC within a few weeks.

Concerning the interpretability of the results, the three decision tree algorithms allow to extract the relative importance of independent variables, thus offering insight into feature importance and possible causal pathways. Table 3 presents these importance metrics as mean percentages.

This study has some limitations. These include that the performance variables are just one set of several possible ones. The irrigation systems were not randomly sampled; hence geographic variations are not representative. In general, the dataset lacks geographical variables, and although we augmented it with monthly rainfall data, more information on these systems would have been desirable. Therefore, we refrain from generalizing across all irrigation systems in Nepal.

Moreover, machine learning treats the independent variables as equal, disregarding any potential causal hierarchy that may exist. However, we are not aware of any quantitative causal model that we could have used for this purpose. Finally, the complex interactions between variables have been discussed extensively in the literature. However, they are not the focus here, hence no new theory, model or explanation is introduced. Instead, we bring empirical evidence to this debate. Therefore, this research contributes to improving the design and successful long-term operation of irrigation systems.

The results should be relatively robust, as we have controlled for many factors that have been suggested as associated with success. However, given that the data only includes Nepalese irrigation systems, we are hesitant to generalize our findings to other Asian countries. Nevertheless, some of the most robust influencing factors may contribute to long-term success in these countries too, see, e.g., [57].

Designing irrigation systems for longevity and good performance – equitable, adequate and predictable water distribution throughout a well-maintained system – is therefore possible even with adverse events. Previous research has made it clear that context [58] is important, and assistance and rules are adapted to the respective local situation [59], while community-based approaches have to be differentiated [60].

Our models suggest which design choices predict success and which do not: If users are allowed to design fair and stable rules for themselves – with arenas to adjust them according to their needs and some assistance from the outside – chances are good that these highly participatory systems survive over long periods. Design of future irrigation systems in Nepal could profit from these insights.

Within a larger context of societal challenges, water scarcity is linked to social instability, violent conflict, and food insecurity [61] with stable institutions recognized as a critical mediating factor [62]. Our findings contribute to this literature by identifying specific design features that predict long-term institutional sustainability and may also buffer communities against the destabilizing effects of water scarcity.

From a policy perspective, the ability to predict which institutional configurations are more resilient may offer practical leverage for conflict prevention and climate adaptation. Our findings suggest that as water stress intensifies across South Asia and beyond, investing in institutional designs that empower local users could prove more effective than infrastructure alone [62].

## Supporting information

S1 File Supplementary Materials. Includes study design, variable descriptions, assumptions, data sources, sampling, protocols and additional analysis.
(DOCX)
