# Supplementary material for: Achieving long-term success in irrigation commons
Source: PLoS One. 2026 Jul 21;21(7):e0353875. doi: 10.1371/journal.pone.0353875 (PMC13387521; doi:10.1371/journal.pone.0353875)
Supplement: S1 File — Includes study design, variable descriptions, assumptions, data sources, sampling, protocols and additional analysis. (DOCX) [file pone.0353875.s001.docx]

# Supplementary Online Materials

### 1. Rainfall data from ERA5

Rainfall data is compiled from the ERA5 data set {Hersbach 2023 #2703}. First, the geographic boundaries of Nepal (80.0884245137, 26.3978980576, 88.1748043151, 30.4227169866) were used to constrain the data which was downloaded via the API by a Python script. The spatial resolution was 0.25-degree x 0.25 degree on a regular latitude-longitude grid which is equivalent to a 250 m resolution. Time resolution is monthly. Matching the irrigation system locations with the latitude and longitude of the ERA5 data was done via Wikipedia and Google maps. For some systems the coordinates where already existing. They were used as checks.

The variable used from this data set is total precipitation. It is defined as “... the accumulated liquid and frozen water, comprising rain and snow, that falls to the Earth's surface. It is the sum of large-scale precipitation and convective precipitation.” The full documentation can be found at <https://cds.climate.copernicus.eu/cdsapp#!/dataset/reanalysis-era5-single-levels-monthly-means?tab=overview>

### 2. List of independent variables

S1 Table S1. Variable names

| **Variable Name** | **Description** | **Institutional?** |
| --- | --- | --- |
| enumapp | Number of users of the entire system (households) | No |
| systarea | Size of the command area (hectares) | No |
| enumusr | Number of users in this village (households) | No |
| length | Length of the canal (m) | No |
| lnthmain | Length of the shared parts of the canal (m) | No |
| intervenTwo | Whether or not it has experienced government intervention (Yes/No). | Yes |
| arenas | Are there arenas being used for the exchange of information about conditions of the resource? (Yes/No) | Yes |
| userate | In the past few years, have takers in this subgroup been taking more or less from the canal than before? (Yes/No) | Yes |
| subalt | Do members also have access to another canal? (Yes/No) | Yes |
| subvar | How big is the difference between the wealthiest and poorest members? (Low/Moderate/High) | Yes |
| subnotOne | Are there any members who spend a lot of time in non-agricultural activities? (Yes/No) | Yes |
| subnotTwo | If YES, what percentage of the group is like that? (1-100, recoded into 6 groups) | Yes |
| avoidhrm | During this time period, have members of this group expended resources (at least their own time) to avoid actions that would harm the structure of the appropriation resource (irrigators who fence their animals or assign someone to watch them so that they do not destroy irrigation works)? (Yes/No) | Yes |
| execapprOne | Is the leader position filled by takers? (Yes/No) | Yes |
| tailadequate | adequacy of water to tailenders (Yes/No) | Performance |
| execapprTwo | If YES, how is the leader selected? (Elections, Government, Other) | Yes |
| endcondpTwo | How well maintained is the headworks? (Good/Fair/Poor) | Performance |
| manage | Do members of this subgroup have the right to participate in the management of this resource? If the members of this subgroup have the authority to make decisions over the use of the resource, then answer yes. This question does not ask the magnitude of the capability, just whether it exists and whether it is being exercised. (Yes/No) | Yes |
| endconddTwo | How well maintained is the canal? (Good/Fair/Poor) | Performance |
| varotime | Is there considerable variation in the flow of units from year to year? From year to year, if the unit yield of the resource fluctuates enough to make a difference in the behavior of the appropriators (e.g., based on seasons, temperature variation, rainfall, etc.), answer "yes." (Yes/No) | Yes |
| penaltyOne | Is there a penalty? (Yes/No) | Yes |
| fair | In your estimation are the rules-in-use: Perceived by members of this subgroup as fair? (Yes/No) | Yes |
| pronurul | Has the subgroup ever tried to change their rules? (Yes/No) | Yes |
| enhance | Have members of this group invested their own labor or other resources in constructing or improving production works? (Yes/No) | Yes |
| endconddOne | Is there any deterioration in the canal? (Yes/No) | Performance |
| tailequity | equity of water to tailenders (Yes/No) | Performance |
| whobuilt | Who constructed (or initiated and directed) the system? (Farmers/Government/Other) | Yes |
| penaltyTwo | Are penalties enforced? (Yes/No) | Yes |
| endcondpOne | Is there any deterioration in the headworks? (Yes/No) | Performance |
| tailpredict | predictability of water to tailenders (Yes/No) | Performance |
| headsameTwo | If YES, does that group/agency operate the headworks alone? (Yes/No) | Yes |
| extrep | Does/do the leader(s), chief executive(s) or administrator(s) report to any external or higher-level authority? (Yes/No) | Yes |
| headsameOne | Are the headworks and canal operated by the same group/agency? (Yes/No) | Yes |
| enfrule | Who enforces the rules? (Members/Officials/Both) | Yes |
| Rainfall January | See SOM, section 1 | No |
| Rainfall February | See SOM, section 1 | No |
| Rainfall March | See SOM, section 1 | No |
| Rainfall April | See SOM, section 1 | No |
| Rainfall May | See SOM, section 1 | No |
| Rainfall June | See SOM, section 1 | No |
| Rainfall July | See SOM, section 1 | No |
| Rainfall August | See SOM, section 1 | No |
| Rainfall   September | See SOM, section 1 | No |
| Rainfall October | See SOM, section 1 | No |
| Rainfall   November | See SOM, section 1 | No |
| Rainfall   December | See SOM, section 1 | No |

### 3. List of learners used in the MLR3 package and their tuning ranges

1. classif.naive_bayes
2. classif.featureless
3. classif.rpart
4. classif.xgboost
5. classif.glmnet (lambda = 0.01)
6. classif.randomForest
7. classif.svm

S1 Table S2. Tuning parameters as implemented in the MLR3-package

| **Algorithm** | **Parameter** | **Min** | **Max** |
| --- | --- | --- | --- |
| Decision Trees | cp | 0.0001 | 0.1 (logscale=TRUE) |
| Decision Trees | minsplit | 1 | 30 |
| Decision Trees | maxdepth | 1 | 30 |
| Decision Trees | minbucket | 1 | 30 |
| GBM | colsample_bynode | 0 | 1 |
| GBM | eta | 0 | 0.7 |
| GBM | nrounds | 50 | 1000 |
| GBM | max_depth | 1 | 13 |
| GBM | lambda | 0 | 2 |
| GBM | gamma | 0.5 | 1 |
| GLM | alpha | 0 | 1 |
| RF | ntree | 50 | 1500 |
| RF | mtry | 1 | 12 |
| SVM | cost | 1e-5 | 1e5 (logscale = TRUE) |
| SVM | gamma | 1e-5 | 1e5 (logscale = TRUE) |

### 4. Best models per algorithm and performance variable

**Round 1**

S1 Table S3. Best models per algorithm and performance variable round 1

| **Performance variable** | **Model** | **Train time** | **Model type** | **Accuracy in %** |
| --- | --- | --- | --- | --- |
| Maintenance of canals (endconddTwo) | GLM not tuned | 1800 | all | 70 |
| Maintenance of headworks (endcondpTwo) | RF not tuned | 600 | institutional factors | 66 |
| Deterioration of headworks (endcondpOne) | GBM tuned | 1800 | all | 81 |
| Deterioration of canals (endconddOne) | RF not tuned | 500 | all | 80 |
| Equity of water at tail | RF not tuned | 600 | institutional factors | 84 |
| Predictability of water at tail | GBM tuned | 400 | institutional factors | 89 |
| Adequacy of water at tail | DT tuned | 600 | all | 77 |

**Round 2**

S1 Table S4. Best models per algorithm and performance variable round 2

| **Performance variable** | **Model** | **Train time** | **Model type** | **Accuracy** |
| --- | --- | --- | --- | --- |
| Maintenance of canals (endconddTwo) | SVM not tuned | 600 | institutional factors | 77 |
| Maintenance of headworks (endcondpTwo) | RF tuned | 300 | all | 58 |
| Deterioration of headworks (endcondpOne) | RF tuned | 600 | all | 80 |
| Deterioration of canals (endconddOne) | RF tuned | 700 | all | 82 |
| Equity of water at tail | DT tuned | 600 | all | 91 |
| Predictability of water at tail | GBM tuned | 600 | institutional factors | 100 |
| Adequacy of water at tail | GBM tuned | 600 | institutional factors | 89 |

### 5. Size correlations

Number of users, round 1, length of main canal, round 1: 0.26***
Number of users, round 1, number of users, round 2: 0.44 ***
Number of users, round 2, length of main canal, round 2: 0.20 **

**Rain correlations:**


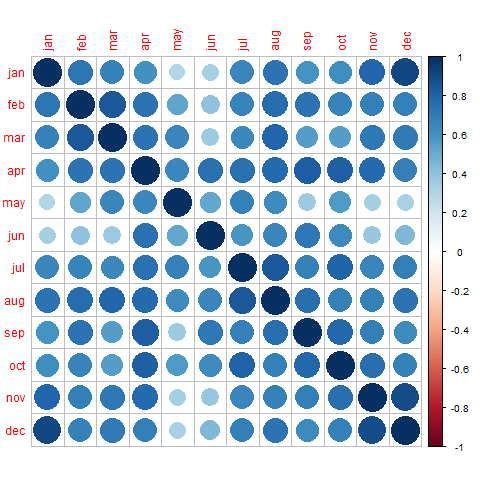


S1 Figure S1. Rain correlations

### 6. Factor importance calculation details

Different measures of importance, like impurity or Gini for random forests are transformed into percentages, so that for each model importance adds up to 1. We report the three variables with the highest mean per performance variable per machine learning model in Table 1 in the main text. As a complement, the most important variable for all round 1 and 2 models, respectively, and the variable with the overall highest count irrespective of mean is added.

Moreover, for each performance variable in R2 predicted from R1 data, a logistic regression was performed. Running an ANOVA on the logistic regression models, the deviance table shows how the models are doing against the null model. A significant drop in a variable’s deviance is tantamount to a high importance of it. Hence, we report the variable with the highest deviance. Finally, we briefly discuss all variables that did not seem to matter for success but have been discussed in the literature for exactly that.

### 7. T-Tests with Bonferroni correction and descriptive statistics

Note: Group 1 are all systems that have been evaluated as “good” or “fair” in respect to the performance variable in each table, as opposed to “poor” performance (= group 2).

S1 Table S5. Maintenance of headworks

| **Variable name** | **Mean group 1** | **Mean group 2** | **T-value** | **DF** | **P-value** |
| --- | --- | --- | --- | --- | --- |
| endcondpOne_2 | 0,917808 | 0,489655 | 8.116 | 214,2071 | 0.00000 |
| tailadequate_2 | 0,630137 | 0,848276 | -3.394 | 112,944 | 0.00024 |
| tailequity_2 | 0,657534 | 0,834483 | -2.768 | 117,4132 | 0.00301 |
| whobuilt_2 | 1,315068 | 1,593103 | -2.920 | 168,805 | 0.00635 |
| endconddTwo_2 | 0,643836 | 0,8 | -2.383 | 123,4858 | 0.01210 |
| subalt_2 | 0,438356 | 0,275862 | 2.344 | 131,4521 | 0.01596 |
| enfrule_2 | 1,958904 | 2,124138 | -2.247 | 185,7336 | 0.04232 |

S1 Table S6. Maintenance of canals

| **Variable name** | **Mean group 1** | **Mean group 2** | **T-value** | **DF** | **P-value** |
| --- | --- | --- | --- | --- | --- |
| tailequity_2 | 0,6 | 0,834356 | -3.220 | 75,78976 | 0.00028 |
| subvar_2 | 2,181818 | 2,533742 | -2.797 | 80,45059 | 0.00242 |
| enfrule_2 | 1,890909 | 2,128834 | -2.940 | 107,2416 | 0.00693 |
| endcondpTwo_2 | 0,527273 | 0,711656 | -2.404 | 85,55608 | 0.01210 |
| tailadequate_2 | 0,654545 | 0,815951 | -2.257 | 79,26114 | 0.01303 |
| intervenTwo_2 | 0,672727 | 0,828221 | -2.209 | 78,5538 | 0.01442 |
| userate_2 | 2,581818 | 3,208589 | -2.501 | 100,337 | 0.01729 |
| endconddOne_2 | 0,909091 | 0,760736 | 2.880 | 137,6593 | 0.01769 |
| arenas_2 | 0,509091 | 0,680982 | -2.225 | 87,382 | 0.02180 |
| execapprTwo_2 | 1,490909 | 1,644172 | -1.972 | 89,28329 | 0.04456 |

S1 Table S7. Deterioration of headworks

| **Variable name** | **Mean group 1** | **Mean group 2** | **T-value** | **DF** | **P-value** |
| --- | --- | --- | --- | --- | --- |
| endcondpTwo_2 | 0,925 | 0,514493 | 7.898 | 214,4708 | 0.00000 |
| endconddOne_2 | 0,6875 | 0,862319 | -2.919 | 129,7714 | 0.00183 |
| subalt_2 | 0,2375 | 0,384058 | -2.312 | 182,9724 | 0.02659 |
| tailadequate_2 | 0,85 | 0,731884 | 2.140 | 193,5423 | 0.04428 |

S1 Table S8. Deterioration of canals

| **Variable name** | **Mean group 1** | **Mean group 2** | **T-value** | **DF** | **P-value** |
| --- | --- | --- | --- | --- | --- |
| endcondpOne_2 | 0,431818 | 0,683908 | -3.023 | 63,14365 | 0.00183 |
| endconddTwo_2 | 0,886364 | 0,712644 | 2.926 | 91,62471 | 0.01769 |
| subalt_2 | 0,477273 | 0,293103 | 2.201 | 61,9287 | 0.02025 |
| manage_2 | 1,727273 | 1,856322 | -1.769 | 56,94507 | 0.04182 |
| penaltyTwo_2 | 0,840909 | 0,689655 | 2.294 | 80,82113 | 0.04611 |

S1 Table S9. Predictability at the tail end

| **Variable name** | **Mean group 1** | **Mean group 2** | **T-value** | **DF** | **P-value** |
| --- | --- | --- | --- | --- | --- |
| varotime_2 | 0,926829 | 0,60452 | 5.832 | 113,2513 | 0.00007 |
| subnotOne_2 | 0,853659 | 0,683616 | 2.578 | 75,03127 | 0.02970 |
| survived | 0,97561 | 0,858757 | 3.261 | 142,8045 | 0.03765 |
| whobuilt_2 | 1,292683 | 1,548023 | -2.346 | 70,07902 | 0.03868 |
| headsameTwo_2 | 0,926829 | 0,983051 | -1.329 | 44,56023 | 0.04767 |

S1 Table S10. Equity at the tail end

| **Variable name** | **Mean group 1** | **Mean group 2** | **T-value** | **DF** | **P-value** |
| --- | --- | --- | --- | --- | --- |
| tailadequate_2 | 0,326531 | 0,905325 | -8.111 | 59,07647 | 0.00000 |
| userate_2 | 1,897959 | 3,384615 | -6.677 | 99,23838 | 0.00000 |
| endconddTwo_2 | 0,55102 | 0,804734 | -3.251 | 66,37783 | 0.00028 |
| endcondpTwo_2 | 0,489796 | 0,715976 | -2.824 | 71,80586 | 0.00301 |
| subnotOne_2 | 0,877551 | 0,668639 | 3.503 | 110,2783 | 0.00417 |
| systarea_2 | 1765,49 | 409,4438 | 1.322 | 48,491 | 0.01713 |
| subvar_2 | 2,22449 | 2,508876 | -2.298 | 74,97722 | 0.01898 |
| enfrule_2 | 1,918367 | 2,112426 | -2.102 | 76,85593 | 0.03487 |

S1 Table S11. Adequacy at the tail end

| **Variable name** | **Mean group 1** | **Mean group 2** | **T-value** | **DF** | **P-value** |
| --- | --- | --- | --- | --- | --- |
| tailequity_2 | 0,326531 | 0,905325 | -8.111 | 59,07647 | 0.00000 |
| userate_2 | 2,204082 | 3,295858 | -4.343 | 84,73745 | 0.00005 |
| endcondpTwo_2 | 0,44898 | 0,727811 | -3.504 | 71,40788 | 0.00024 |
| endconddTwo_2 | 0,612245 | 0,786982 | -2.267 | 68,52565 | 0.01303 |
| extrep_2 | 0,510204 | 0,337278 | 2.139 | 74,28252 | 0.02782 |
| execapprThree_2 | 0,653061 | 0,792899 | -1.853 | 69,09083 | 0.04337 |
| endcondpOne_2 | 0,755102 | 0,597633 | 2.166 | 86,86704 | 0.04428 |
| systarea_2 | 1597,776 | 458,071 | 1.120 | 48,71069 | 0.04553 |

S1 Table S12. Survival

| **Variable name** | **Mean group 1** | **Mean group 2** | **T-value** | **DF** | **P-value** |
| --- | --- | --- | --- | --- | --- |
| penaltyOne_2 | 0 | 0,380208 | -10.82 | 191 | 0.00010 |
| resourid | 52189,08 | 51668,04 | 4.875 | 44,01053 | 0.00061 |
| whobuilt_2 | 1,076923 | 1,557292 | -5.156 | 52,31909 | 0.00116 |
| varotime_2 | 0,923077 | 0,630208 | 4.596 | 49,88807 | 0.00285 |
| avoidhrm_2 | 0,346154 | 0,125 | 2.254 | 28,24787 | 0.00302 |
| subvar_2 | 2,153846 | 2,484375 | -2.157 | 32,42409 | 0.03448 |
| predThreevar_2 | 1,038462 | 1,208333 | -3.510 | 60,02802 | 0.03765 |
| tailpredict_2 | 0,961538 | 0,791667 | 3.510 | 60,02802 | 0.03765 |
| lnthmain_2 | 6452,615 | 4523,057 | 1.501 | 28,23776 | 0.04673 |

S1 Table S13. Descriptive statistics for explanatory variables

| **Variable names** | **n** | **mean** | **sd** | **min** | **max** |
| --- | --- | --- | --- | --- | --- |
| resourid | 218 | 51730.18 | 735.12 | 50506 | 52427 |
| arenas_1 | 218 | 0.82 | 0.39 | 0 | 1 |
| avoidhrm_1 | 218 | 0.56 | 0.5 | 0 | 1 |
| endconddOne_1 | 218 | 0.29 | 0.46 | 0 | 1 |
| endconddTwo_1 | 218 | 2.06 | 0.63 | 1 | 3 |
| endcondpOne_1 | 218 | 0.24 | 0.43 | 0 | 1 |
| endcondpTwo_1 | 218 | 2.06 | 0.65 | 1 | 3 |
| enumapp_1 | 218 | 684.92 | 2846.24 | 1 | 32000 |
| enumusr_1 | 218 | 694.54 | 2841.91 | 5 | 32000 |
| execapprOne_1 | 218 | 0.76 | 0.43 | 0 | 1 |
| execapprTwo_1 | 218 | 1.24 | 0.43 | 1 | 2 |
| extrep_1 | 218 | 0.12 | 0.33 | 0 | 1 |
| enfrule_1 | 218 | 1.9 | 0.3 | 1 | 2 |
| enhance_1 | 218 | 0.8 | 0.4 | 0 | 1 |
| fair_1 | 218 | 0.79 | 0.41 | 0 | 1 |
| headsameOne_1 | 218 | 0.82 | 0.38 | 0 | 1 |
| headsameTwo_1 | 218 | 0.82 | 0.38 | 0 | 1 |
| intervenTwo_1 | 218 | 0.61 | 0.49 | 0 | 1 |
| length_1 | 218 | 10526.61 | 16599.95 | 100 | 112500 |
| lnthmain_1 | 218 | 3980.85 | 4682.59 | 100 | 32780 |
| manage_1 | 218 | 1.6 | 0.49 | 1 | 2 |
| penaltyOne_1 | 218 | 0.8 | 0.4 | 0 | 1 |
| penaltyTwo_1 | 218 | 0.67 | 0.47 | 0 | 1 |
| pronurul_1 | 218 | 0.72 | 0.45 | 0 | 1 |
| subalt_1 | 218 | 0.32 | 0.47 | 0 | 1 |
| subnotOne_1 | 218 | 0.24 | 0.43 | 0 | 1 |
| subnotTwo_1 | 218 | 1.24 | 0.43 | 1 | 2 |
| subvar_1 | 218 | 2.27 | 0.82 | 1 | 3 |
| systarea_1 | 218 | 659.97 | 2474.24 | 1 | 27844 |
| tailpredict_1 | 218 | 0.8 | 0.4 | 0 | 1 |
| tailadequate_1 | 218 | 0.4 | 0.49 | 0 | 1 |
| tailequity_1 | 218 | 0.72 | 0.45 | 0 | 1 |
| userate_1 | 218 | 2.41 | 0.69 | 1 | 3 |
| varotime_1 | 218 | 0.72 | 0.45 | 0 | 1 |
| whobuilt_1 | 218 | 1.28 | 0.54 | 1 | 3 |
| arenas_2 | 218 | 0.64 | 0.48 | 0 | 1 |
| avoidhrm_2 | 218 | 0.15 | 0.36 | 0 | 1 |
| endconddOne_2 | 218 | 0.8 | 0.4 | 0 | 1 |
| endconddTwo_2 | 218 | 1.69 | 0.85 | 1 | 3 |
| endcondpOne_2 | 218 | 0.63 | 0.48 | 0 | 1 |
| endcondpTwo_2 | 218 | 1.96 | 0.84 | 1 | 3 |
| enumapp_2 | 218 | 463.02 | 964.16 | 9 | 6000 |
| enumusr_2 | 218 | 505.33 | 1194.44 | 7 | 10000 |
| execapprOne_2 | 218 | 0.95 | 0.21 | 0 | 1 |
| execapprTwo_2 | 218 | 1.61 | 0.49 | 1 | 2 |
| execapprThree_2 | 218 | 0.76 | 0.43 | 0 | 1 |
| extrep_2 | 218 | 0.38 | 0.49 | 0 | 1 |
| enfrule_2 | 218 | 2.07 | 0.57 | 1 | 3 |
| enhance_2 | 218 | 0.94 | 0.24 | 0 | 1 |
| fair_2 | 218 | 0.68 | 0.47 | 0 | 1 |
| headsameOne_2 | 218 | 0.91 | 0.28 | 0 | 1 |
| headsameTwo_2 | 218 | 0.97 | 0.16 | 0 | 1 |
| intervenTwo_2 | 218 | 0.79 | 0.41 | 0 | 1 |
| lnthmain_2 | 218 | 4753.19 | 4648.09 | 100 | 27000 |
| manage_2 | 218 | 1.83 | 0.38 | 1 | 2 |
| penaltyOne_2 | 218 | 0.33 | 0.47 | 0 | 1 |
| penaltyTwo_2 | 218 | 0.72 | 0.45 | 0 | 1 |
| pronurul_2 | 218 | 0.37 | 0.48 | 0 | 1 |
| predThreevar_2 | 218 | 1.19 | 0.39 | 1 | 2 |
| subalt_2 | 218 | 0.33 | 0.47 | 0 | 1 |
| subnotOne_2 | 218 | 0.72 | 0.45 | 0 | 1 |
| subvar_2 | 218 | 2.44 | 0.75 | 1 | 3 |
| systarea_2 | 218 | 714.24 | 3516.68 | 2 | 49000 |
| tailpredict_2 | 218 | 0.81 | 0.39 | 0 | 1 |
| tailadequate_2 | 218 | 0.78 | 0.42 | 0 | 1 |
| tailequity_2 | 218 | 0.78 | 0.42 | 0 | 1 |
| userate_2 | 218 | 3.05 | 1.69 | 1 | 5 |
| varotime_2 | 218 | 0.67 | 0.47 | 0 | 1 |
| whobuilt_2 | 218 | 1.5 | 0.71 | 1 | 3 |
| survived | 218 | 0.88 | 0.32 | 0 | 1 |
| lat | 218 | 27.8 | 0.41 | 26.5 | 29.5 |
| lon | 218 | 84.23 | 1.26 | 80.75 | 88 |
| jan_tp | 218 | 0 | 0 | 0 | 0.01 |
| feb_tp | 218 | 0 | 0 | 0 | 0 |
| mar_tp | 218 | 0 | 0 | 0 | 0 |
| apr_tp | 218 | 0 | 0 | 0 | 0.01 |
| may_tp | 218 | 0 | 0 | 0 | 0 |
| jun_tp | 218 | 0.01 | 0 | 0 | 0.02 |
| jul_tp | 218 | 0.02 | 0.01 | 0.01 | 0.04 |
| aug_tp | 218 | 0.01 | 0 | 0.01 | 0.03 |
| sep_tp | 218 | 0.02 | 0 | 0.01 | 0.03 |
| oct_tp | 218 | 0 | 0 | 0 | 0 |
| nov_tp | 218 | 0 | 0 | 0 | 0 |
| dec_tp | 218 | 0 | 0 | 0 | 0 |

### Map of irrigation systems


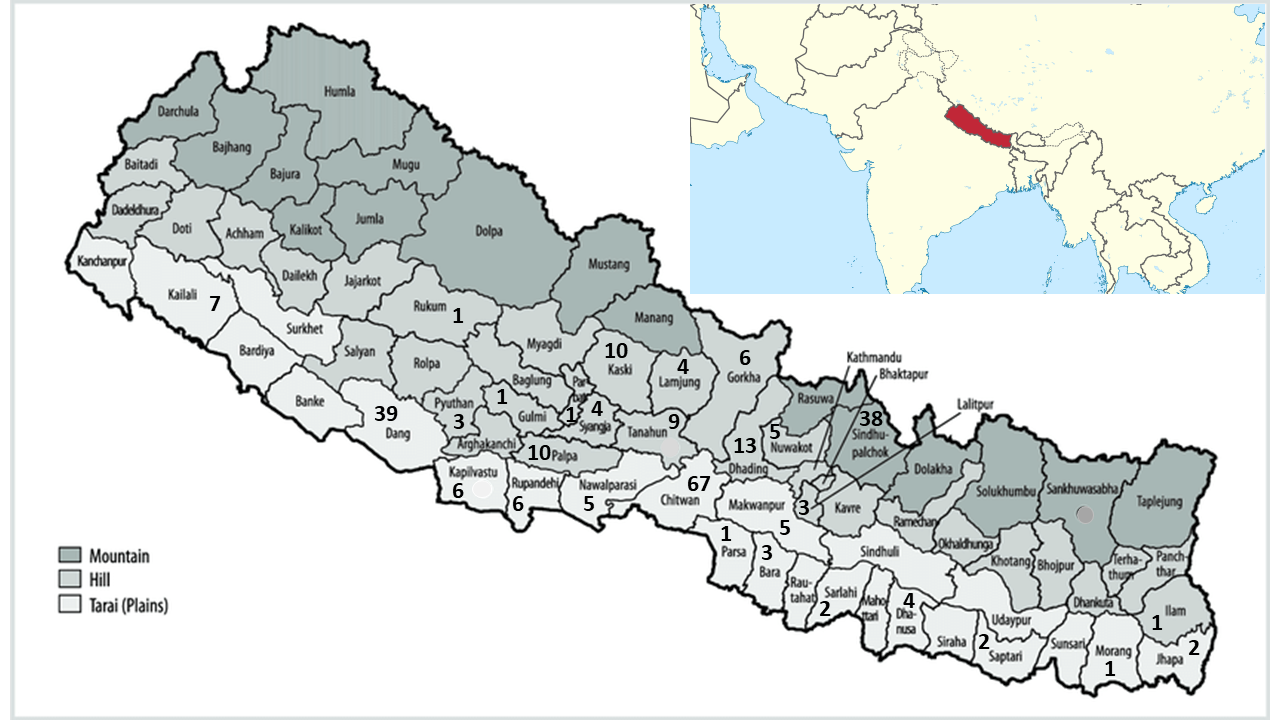


S1 Figure S2. Locations of irrigation systems (number designates number of systems per district), modified after <https://www.researchgate.net/figure/Map-of-Nepal-showing-district-boundaries-ecological-zones-and-research-sites-Map-by_fig1_320063047>, which has been published under the Creative Commons Attribution 4.0 International license. The map in the right-hand corner is from <https://upload.wikimedia.org/wikipedia/commons/9/91/Nepal_in_Asia_%28-mini_map_-rivers%29.svg> and has been published under the Creative Commons Attribution 3.0 license

The systems in the first and second round were the same, and therefore the sampling is the same.

**8.1 Locating the original systems for the second round**

To make the datasets comparable, it was necessary to systematically locate the original system. For each irrigation system in the original database, the enumerators used the location and village name from the original survey to find it. If they could locate the canal but not the village, they chose the village closest to the head and made note of it. If the village had split since the first survey, they chose the one closest to the head and made note of it. If two villages had combined, they made note of it. If the canal had split, they chose the village that was using the named water source for irrigation. They walked the length of the canal to inspect damage, and to examine the headworks. Where the physical infrastructure was not recognizable because, for instance, a road had been built on top of it, the canal's location was verified by interviewing those in the area.

### Short description of the data pre-processing steps

The data preprocessing steps for the data were as follows:

- Trim all character strings
- Make all columns numeric
- Convert all missing fields to NA
- Rename variables
- Combine (merge) round 1 and round 2 data
- Recode factor variables from numeric back to factor

### Data set, data quality and assumptions

As two well-known datasets, these sources have been discussed in full detail elsewhere (e.g. [8, 27, 43]). Here we reproduce a subset of that material that is relevant to the results in this paper. The information for the variables comes from the Nepal Irrigation Institutions and Systems (NIIS) database maintained by the Ostrom Workshop in Political Theory and Policy Analysis at Indiana University in Bloomington and online (https://ulrichfrey.eu/en/niis/).

This resource documents institutional and organizational characteristics of 263 community-managed irrigation systems across Nepal. Each system is described using 566 recorded variables that cover multiple domains, including geographic context (such as location in the Terai versus hill regions), engineering and infrastructural elements (for example, the presence of headworks), biophysical characteristics (such as system size), social composition (including measures like ethnic heterogeneity), and institutional arrangements (such as rules for water distribution, monitoring practices, and collective decision-making procedures).

One variable of particular interest has been the type of management: 21 cases (8%) are managed by the government (AMIS, *agency-managed irrigation systems*), 208 cases (79%) are managed by users (FMIS, *farmer-managed irrigation systems*), 28 cases (11%) are managed by both (JMIS, *joint management irrigation systems*), and 6 systems (2%) are unknown.

The dataset is considered highly reliable. Every case was independently coded by multiple researchers, and gaps in the original records were addressed through follow-up field visits in Nepal. As a result, no observations had to be dropped from the analysis. Missing information is minimal overall: out of 9,516 total observations (244 non-duplicated systems of 263 case studies multiplied by 39 variables used here), only about five percent of entries are absent. Overall, the follow-up sample (Round 2) is complete because it contains all questions asked in Round 1 and as comparable as possible.

It is assumed that problems of measurement precision do not affect this dataset because it contains only *subjective* assessments. We assume that biases introduced by comparability problems of the two datasets are mitigated to some extent because tail-end responses were used whenever there was ambiguity about whether the respondents were located at the head or tail of the original system.

Since the main goal of the second round of data collection was to revisit the sites of the original data set, which is not representative, no effort was taken to go beyond the sampling methods of the original dataset.

The comparability of subjective assessments cannot be guaranteed, but there is nothing in the data to lead us to assume that any of this variability is significant: first, the survey instrument was almost identical in both rounds. Except for some minor modifications, the original questions were kept, since the goal of round 2 was a replication. Second, the author who gave the instructions to the fieldworkers is intimately familiar with the original work of Elinor Ostrom and her colleagues on the NIIS. They gained this familiarity, in part, by interviewing the researchers who conducted the original study. This means also that nuances and the broader context, as well as the core concepts could be explained to the fieldworkers. Third, we provide descriptive statistics on all variables from round 1 and 2 – if there was a bias in answers, this would show up there. Fourth, whenever variables have been combined or collapsed, we have taken great care to ensure comparability.

To ensure completeness, we assume that in those cases where systems could not be located and where respondents could not be identified, the systems were no longer in use in the second round. Finally, we assume that assessments made by enumerators are not significantly sensitive to different enumerators, since they were trained following the same protocol. This protocol is described in section 12 of the SOM titled “Protocol for enumerators”.

### Mapping of design principles and performance variables

S1 Table S14. Mapping of design principles to performance variables

| **Performance variable** | **Design principle** |
| --- | --- |
| *Maintenance of headworks* | Graduated sanctions,  Collective-choice arrangements |
| *Maintenance of canals* | Collective-choice arrangements |
| *Deterioration of headworks* | Minimal recognition of rights to organize |
| *Deterioration of canals* | Minimal recognition of rights to organize, Collective-choice arrangements |
| *Predictability of water at tail* | Collective-choice arrangements,  Graduated sanctions |
| *Adequacy of water at tail* | Minimal recognition of rights to organize, Collective-choice arrangements |
| *Equity of water at tail* | Collective-choice arrangements,  Graduated sanctions |
| *Survival* | Minimal recognition of rights to organize, Collective-choice arrangements |

### Protocol for enumerators

All enumerators were trained for two to three weeks before administering the survey instrument. Each enumeration team consisted of two members. All the enumerators had college education and at least one member of each team was a civil engineer. All audio was recorded while administering the survey, unless permission was denied by respondents. No identifying information was recorded in the audio. Each instrument was filled out based on multiple responses and therefore these responses could not be traced to individual respondents. I trained the enumerators to inspect the canals and classify any damage they saw, as well as the level of water and the amount of siltation.

For each irrigation system in the original database, the enumerators used the location and village name from the original survey to find it. If they could locate the canal but not the village, they chose the village closest to the head and made note of it. If the village had split since the first survey, they chose the one closest to the head and made note of it. If two villages had combined, they made note of it. If the canal had split, they chose the village that was using the named water source for irrigation. They walked the length of the canal to inspect damage, and to examine the headworks. Where the physical infrastructure was not recognizable because, for instance, a road had been built on top of it, the canal's location was verified by interviewing those in the area.

The unit of analysis is the group of users. The members are the primary respondents. The respondents were chosen as follows: For each canal, identify the same set of users in the village if noted in the survey. Otherwise, treat the group of current users as the group of respondents. From this group, choose at least 3 respondents, such that one is a poorer user. A poorer user is one who appears to have fewer assets and smaller landholdings. Choose one who appears to be knowledgeable about the canal and choose one who was the first to speak to them. Speak to more users if further information is required. In case of canals that no longer exist, look for older users who had lived there continuously. Among them, find former users. Choose one as the primary respondent. Overall, if you receive multiple conflicting answers to a question, try to resolve it by speaking to more users. Use reasoned judgment and instinct when aggregating the answers.

Thus, enumerators interviewed at least three users: the first user that was willing to answer questions, one who was regarded as being knowledgeable about the canal, and a poorer looking user based on their material condition (house, appearance, size of land).

Enumerators aggregated subjective responses as follows: any occurrence of a poor assessment resulted in an aggregate of poor, and for the others, whether satisfactory or good was more frequent across respondents determined the coding. Enumerators then returned to Kathmandu and entered the data using a custom web-based form (using MySQL and PHP).

Finally, in cases where this process for aggregating subjective disagreements was inconclusive, the entire team discussed the recorded discussions to finalize the encoding. Where this could not be determined, the response was marked missing.

### Correlation matrices of key predictors for the performance variables

Correlations are separated by type, i.e. binary and factor variables and by round. For variable names, see Table S1. Variables headsameTwo_1 and arenfreq_2 have been deleted due to too many missing values.

*S1 Figure S3. Correlation matrix for all binary key predictors for round 1.*


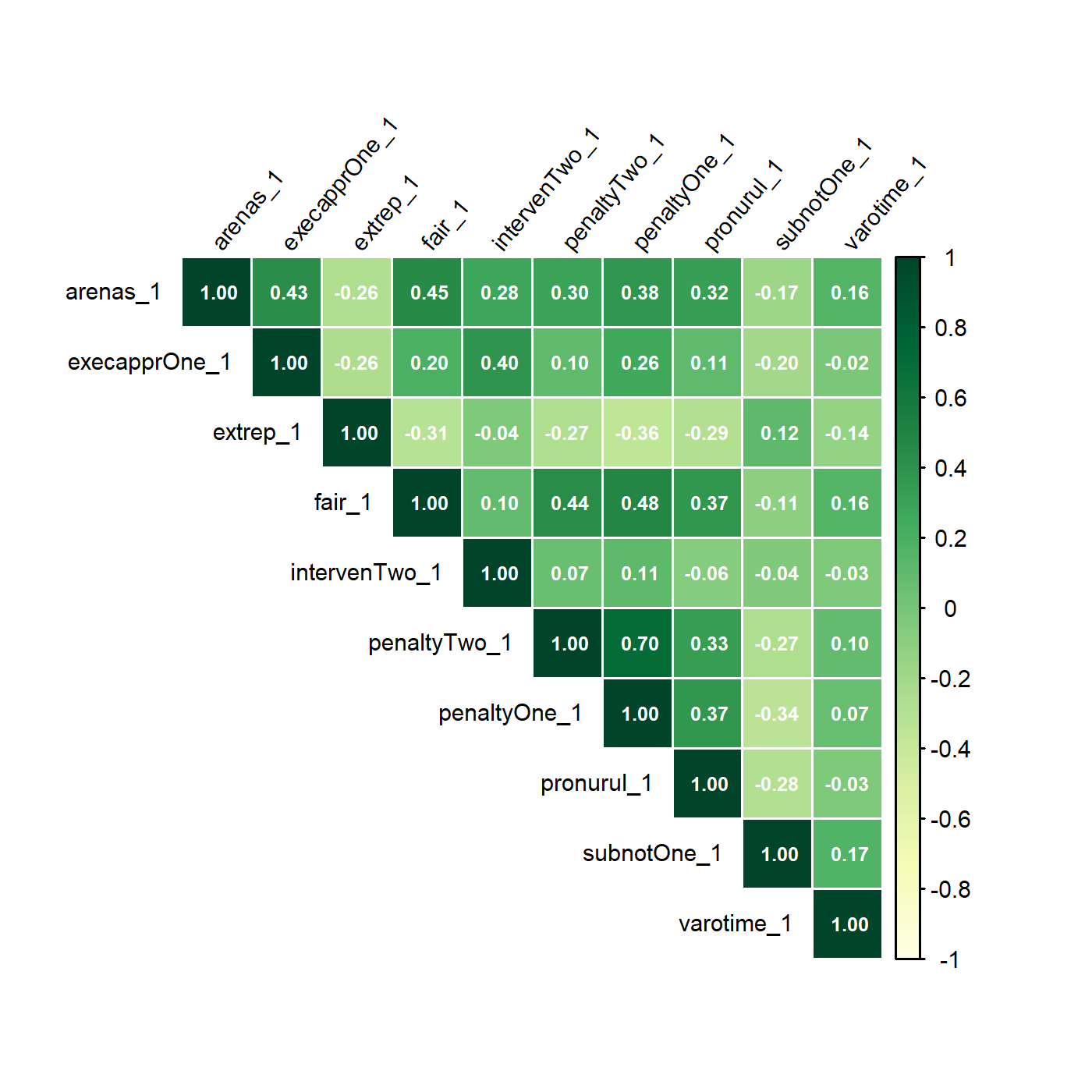


*S1 Figure S4. Correlation matrix for all factorial key predictors for round 1.*

*
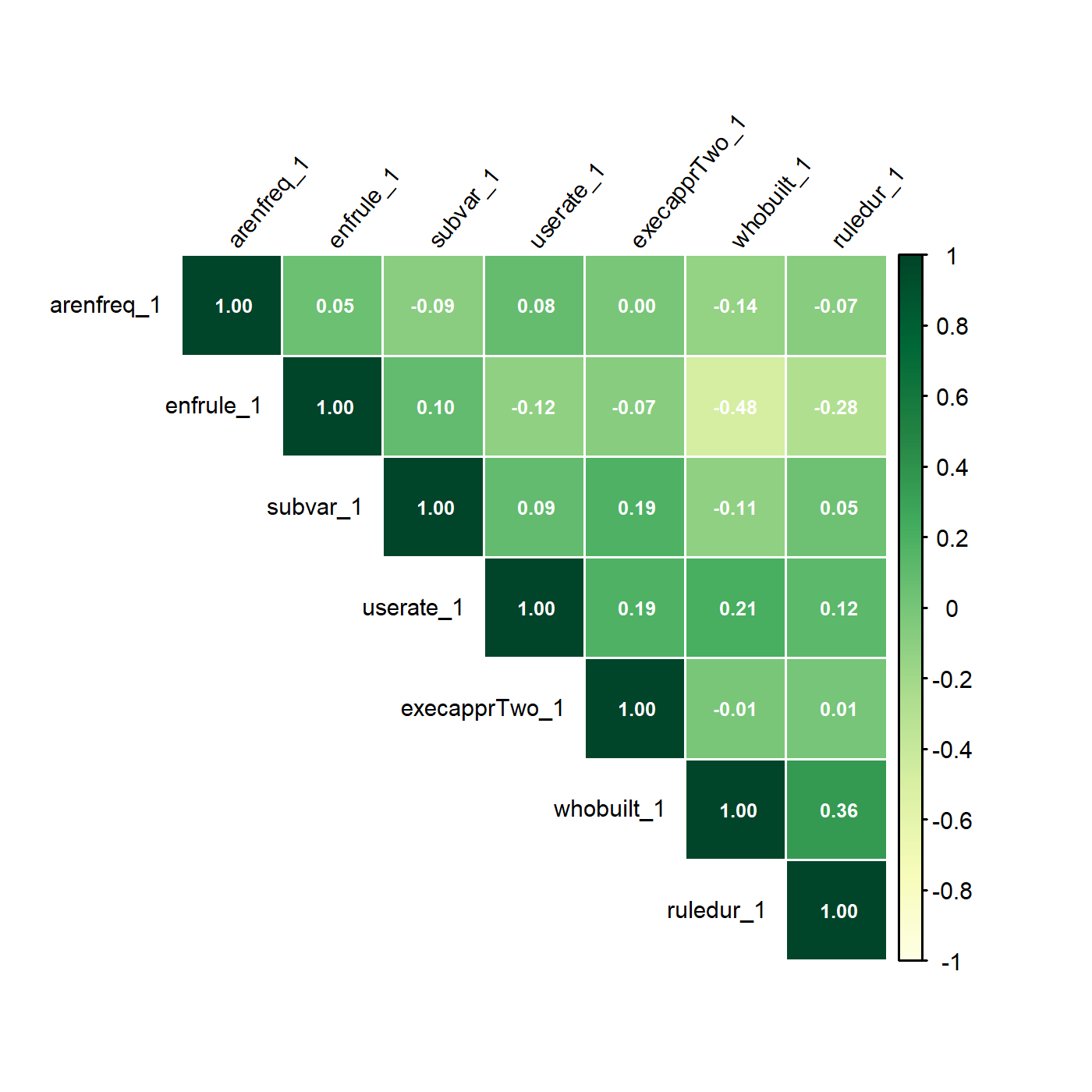
*

*S1 Figure S5. Correlation matrix for all binary key predictors for round 2.*

*
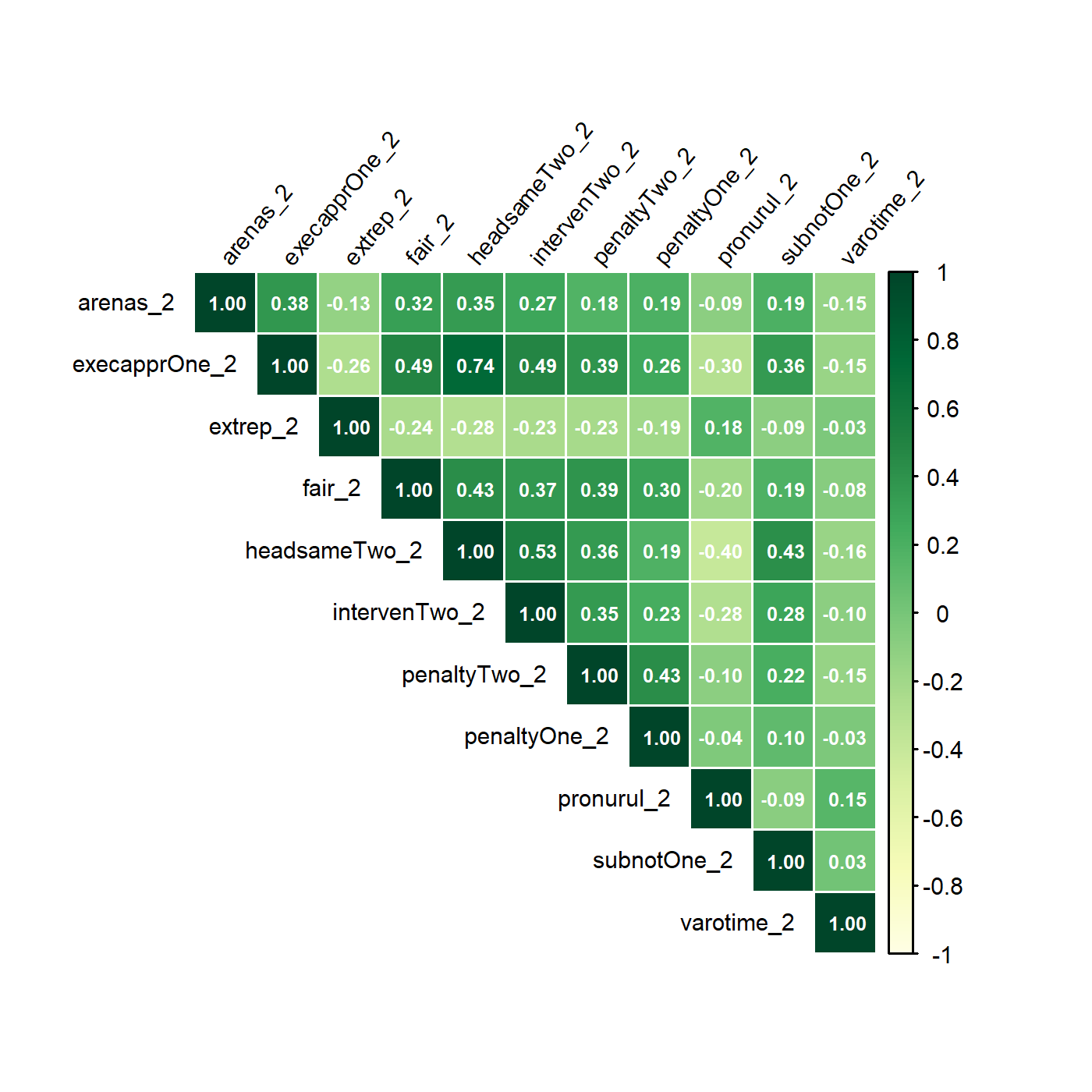
*

*S1 Figure S6. Correlation matrix for all factorial key predictors for round 2.*

*
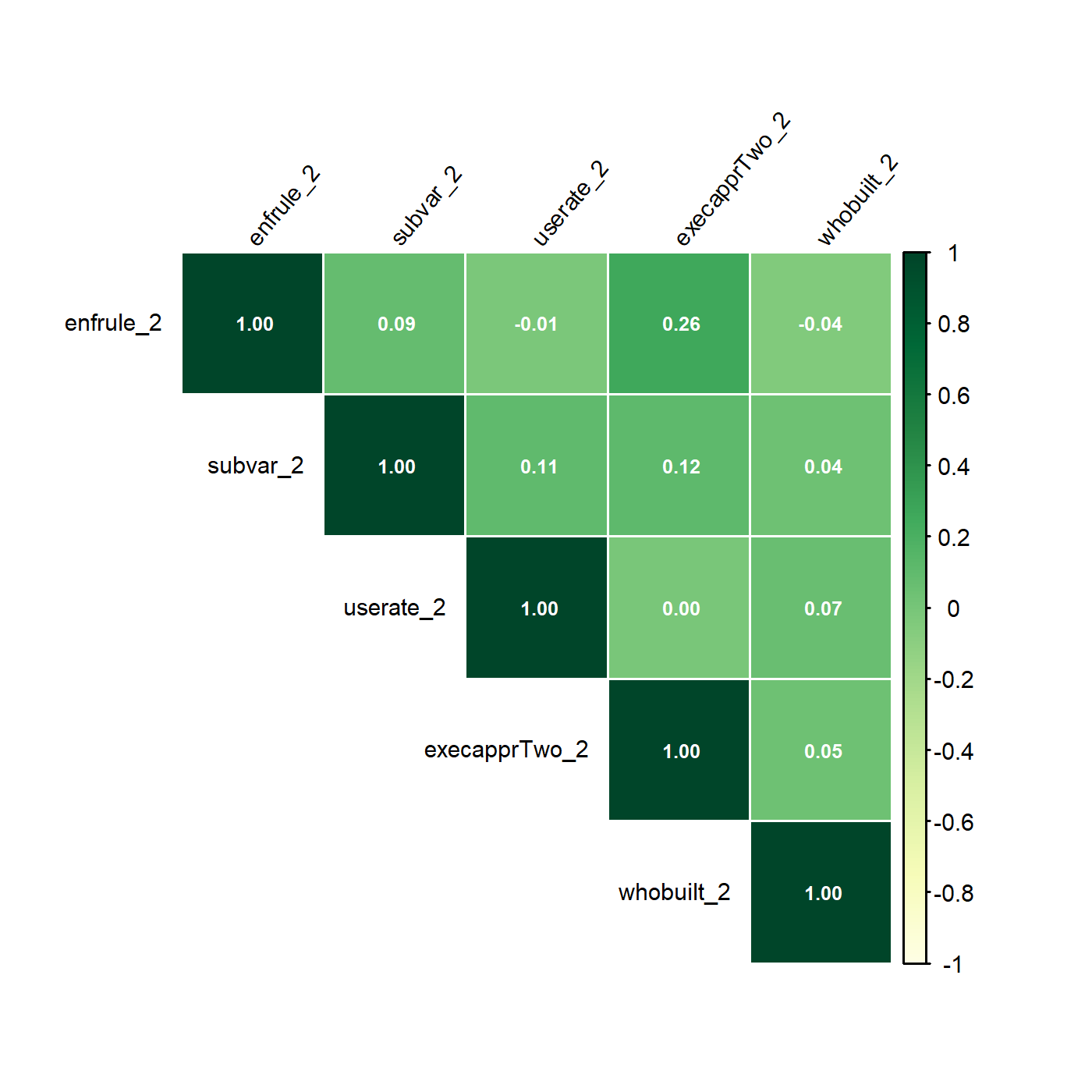
*
